# On the Use of Graphene Nanosheets for Drug Delivery: A Case Study of Cisplatin and Some of Its Analogs

**DOI:** 10.3390/pharmaceutics15061640

**Published:** 2023-06-01

**Authors:** Mahmoud A. A. Ibrahim, Manar H. A. Hamad, Amna H. M. Mahmoud, Gamal A. H. Mekhemer, Shaban R. M. Sayed, Mohamed K. Abd El-Rahman, Peter A. Sidhom, Eslam Dabbish, Tamer Shoeib

**Affiliations:** 1 Computational Chemistry Laboratory, Chemistry Department, Faculty of Science, Minia University, Minia 61519, Egypt; m.hamad@compchem.net (M.H.A.H.); a.mahmoud@compchem.net (A.H.M.M.); gmekhemer@mu.edu.eg (G.A.H.M.); 2 School of Health Sciences, University of KwaZulu-Natal, Westville, Durban 4000, South Africa; 3 Department of Botany and Microbiology, College of Science, King Saud University, P.O. Box 2455, Riyadh 11451, Saudi Arabia; shmohamed@ksu.edu.sa; 4 Department of Chemistry and Chemical Biology, Harvard University, 12 Oxford Street, Cambridge, MA 02138, USA; kabdelazim@gmwgroup.harvard.edu; 5 Department of Pharmaceutical Chemistry, Faculty of Pharmacy, Tanta University, Tanta 31527, Egypt; peter.ayoub@pharm.tanta.edu.eg; 6 Department of Chemistry, The American University in Cairo, New Cairo 11835, Egypt; emoustafa@aucegypt.edu

**Keywords:** graphene, anti-cancer drug, adsorption process, DFT

## Abstract

Graphene (GN) nanosheets have been widely exploited in biomedical applications as potential nanocarriers for various drugs due to their distinct physical and chemical properties. In this regard, the adsorption behavior of cisplatin (*cis*PtCl_2_) and some of its analogs on a GN nanosheet was investigated in perpendicular and parallel configurations by using density functional theory (DFT). According to the findings, the most significant negative adsorption energies (*E*_ads_) within the *cis*PtX_2_⋯GN complexes (where X = Cl, Br, and I) were observed for the parallel configuration, with values up to –25.67 kcal/mol at the H@GN site. Within the perpendicular configuration of the *cis*PtX_2_⋯GN complexes, three orientations were investigated for the adsorption process, namely, X/X, X/NH_3_, and NH_3_/NH_3_. The negative *E*_ads_ values of the *cis*PtX_2_⋯GN complexes increased with the increasing atomic weight of the halogen atom. The Br@GN site showed the largest negative *E*_ads_ values for the *cis*PtX_2_⋯GN complexes in the perpendicular configuration. The Bader charge transfer outcomes highlighted the electron-accepting properties of *cis*PtI_2_ within the *cis*PtI_2_⋯GN complexes in both configurations. The electron-donating character of the GN nanosheet increased as the electronegativity of the halogen atom increased. The band structure and density of state plots revealed the occurrence of the physical adsorption of the *cis*PtX_2_ on the GN nanosheet, which was indicated by the appearance of new bands and peaks. Based on the solvent effect outlines, the negative *E*_ads_ values generally decreased after the adsorption process in a water medium. The recovery time results were in line with the *E*_ads_ findings, where the *cis*PtI_2_ in the parallel configuration took the longest time to be desorbed from the GN nanosheet with values of 61.6 × 10^8^ ms at 298.15 K. The findings of this study provide better insights into the utilization of GN nanosheets in drug delivery applications.

## 1. Introduction

A graphene (GN) nanosheet is a flat monolayer of *sp*^2^-hybrid carbon atoms with a tightly packed two-dimensional hexagonal lattice with a zero electronic band gap at the Fermi level [1]. GN nanosheets have unique characteristics, including robust charge carrier mobility, lower toxicity, and a large surface area [2,3]. GN-based materials have been utilized in a wide range of applications, including energy storage, sensors, and photodetectors [4,5,6,7,8,9,10,11]. Recently, the potential uses of GN-based materials in the biomedical field, including in cancer therapeutics, biosensors, bioimaging, and drug/gene delivery, have garnered tremendous attention [12,13,14,15,16,17,18,19]. Within the drug delivery application context, Liu et al. demonstrated the potentiality of GN-based materials for delivering water-insoluble anticancer drugs [14]. Subsequently, the utilization of GN nanosheets to deliver various anticancer drugs, such as cladribine, 6-Mercaptopurine, 5-Fluorouracil, and *β*-lapachone drugs, was investigated [20,21,22].

Metal-based compounds have long been thought to have therapeutic potential because metals exhibit superior properties, such as reactivity toward organic substrates, redox activity, and variable coordination modes [23]. In 1978, Cisplatin (*cis*PtCl_2_), a platinum-metal-based drug with the molecular formula *cis*-[PtCl_2_(NH_3_)_2_], was approved by the U.S. Food and Drug Administration (FDA) and has since been commonly used as an effective drug against lung, ovarian, and colorectal cancers [24,25,26,27,28,29,30]. The enormous clinical success of the *cis*PtCl_2_ drug has sparked an intensive search for platinum analogs with possibly superior biological and pharmacological properties. Examples of such analogs were prepared by replacing the two chloride atoms of the *cis*PtCl_2_ with different halides, including bromide and iodide atoms [31,32]. Intriguingly, *cis*-[PtBr_2_(NH_3_)_2_] (*cis*PtBr_2_) and *cis*-[PtI_2_(NH_3_)_2_] (*cis*PtI_2_) exhibited remarkable biological characteristics relative to the *cis*PtCl_2_ drug [31,32]. However, generally, platinum-based chemotherapy has serious side effects caused by its low specificity and non-selectivity, resulting in systemic toxicities that severely limit its efficacy [33,34]. One of the potential remedies for these side effects is utilizing a nanocarrier that can offer a better-guided delivery form that increases the percentage of the drug that reaches the target cancerous cells out of the administered dose. This, in turn, decreases the systemic dose and increases the therapeutic outcomes [35,36]. In this regard, GN-based materials were employed as effective nanocarriers for the *cis*PtCl_2_ drug [37,38,39,40]. More than one theoretical study was conducted with the aim of characterizing the nature of the interactions between drugs, including cisplatin, and non-drug biological adsorbents with GN and GN-oxide surfaces [11,37,38]. However, the potential of GN nanosheets for delivering the *cis*PtBr_2_ and *cis*PtI_2_ analogs has not yet been investigated.

**Figure 1:**
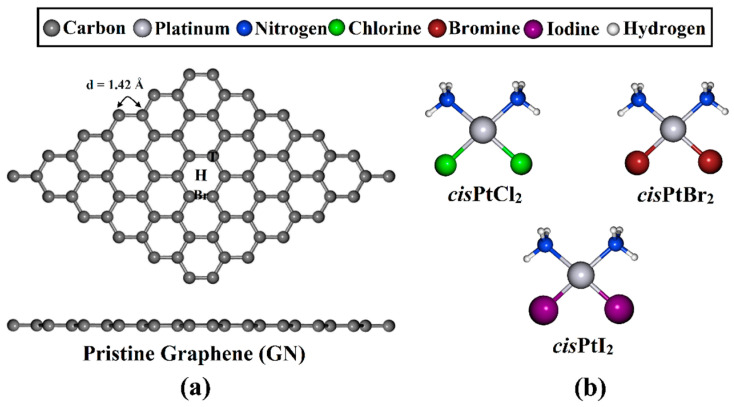
(**a**) Top and side representations of the optimized GN nanosheet with three adsorption sites, namely, the top (T), hollow (H), and bridge (Br) sites, and (**b**) the structures of *cis*PtX_2_ (where X = Cl, Br, and I).

In the current study, the utilization of GN nanosheets as a drug delivery system for the anticancer drug *cis*PtCl_2_ and its analogs was investigated (Figure 1). The adsorption behavior of the *cis*PtX_2_ (where X = Cl, Br, and I) molecules on a GN nanosheet was systematically investigated and comparatively assessed via various DFT calculations. To better understand the adsorption process of the *cis*PtX_2_⋯GN complexes, adsorption of the *cis*PtX_2_ on the GN nanosheet was conducted at different adsorption sites in perpendicular and parallel configurations. Geometric optimizations were performed for all complexes, followed by adsorption energy calculations. Based on the relaxed structures, post-analyses, including Bader charge, density of states (DOS), and band structure, were executed for the most favorable *cis*PtX_2_⋯GN complexes. Furthermore, the solvent effect and recovery time were evaluated for the most favorable configurations. This study provides an understanding of the adsorption energetics, binding relationships, regioselectivity, and electron donor/acceptor sites of the *cis*PtX_2_ molecules and GN nanosheet. This can further support the informative design of GN nanocarriers that can better suit *cis*PtX_2_ drug delivery.

## 2. Computational Methods

All computations for the adsorption of *cis*PtX_2_ on GN were performed with the DFT method [41,42] as implemented in Quantum ESPRESSO 6.4.1 code [43,44]. The Perdew–Burke–Ernzerhof method within the generalized gradient approximation was applied to describe the exchange-correlation functional of the electronic interactions [45]. To denote the electron–ion interactions, the ultrasoft pseudopotential was utilized [46]. Grimme’s DFT-D2 method was adopted to correct the dispersion interaction [47]. The cutoffs of the optimized kinetic energy and the charge density were set to 40 and 400 Ry, respectively. For all calculations, the thresholds for force and energy convergence were chosen at 10^−4^ eV/Å and 10^−5^ eV, respectively. The first Brillouin zone was sampled depending on Monkhorst–Pack grids as 4 × 4 × 1 and 8 × 8 × 1 *k*-points for the geometry optimization and the density of states calculations, respectively. Moreover, the Marzari–Vanderbilt smearing method [48] was applied with a Gaussian spreading value of 10^−4^ Ry. A vacuum region of 20 Å was generated to separate artificially periodic cells along the *z*-direction of the GN surface. A 6 × 6 × 1 supercell involving 72 carbon atoms was designed to investigate the adsorption process.

**Figure 2:**
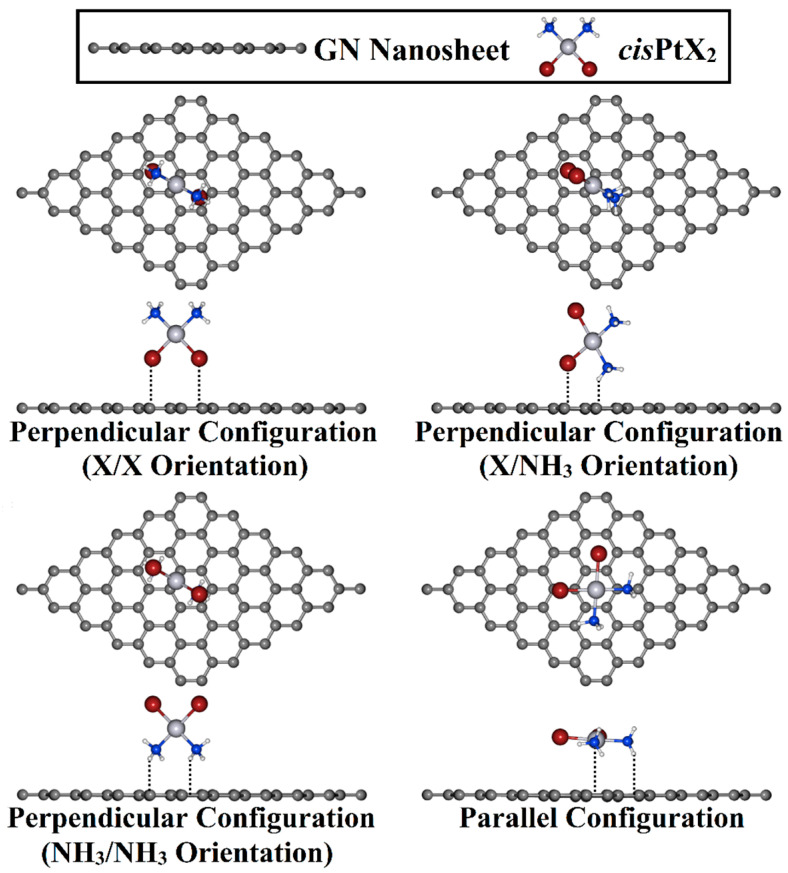
Top and side illustrations of the adsorption of the *cis*PtX_2_ (where X = Cl, Br, and I) on the GN nanosheet in perpendicular and parallel configurations.

Adsorption of *cis*PtX_2_ over the GN nanosheet was investigated in perpendicular and parallel configurations (Figure 2). For the perpendicular configuration, three different orientations for the *cis*PtX_2_, namely, X/X, X/NH_3_, and NH_3_/NH_3_, were considered for the adsorption process on the GN nanosheet. Based on the optimized geometries, the adsorption energy (*E*_ads_) was estimated based on the following formula:

(1) $$E_{ads}=E_{{cisPtX}_{2}\cdots GN}-({E_{{cisPtX}_{2}}+E}_{GN})$$

where $E_{{cisPtX}_{2}\cdots GN}$, $E_{{cisPtX}_{2}}$, and $E_{GN}$ represent the energies of the complex, the adsorbed *cis*PtX_2_, and the GN nanosheet, respectively. For a more detailed examination of the adsorption process of *cis*PtX_2_ molecules on the GN nanosheet, frontier molecular orbital (FMO) calculations were performed. Based on the FMO analyses, the energies of the highest occupied molecular orbitals (*E*_HOMO_) and lowest unoccupied molecular orbitals (*E*_LUMO_) were computed for the most favorable relaxed *cis*PtX_2_⋯GN complexes. The energy gap (*E*_gap_) was calculated according to the following formula:

(2) $$E_{gap}=E_{LUMO}-E_{HOMO}$$

In addition, Bader charge analysis [49,50] was applied to determine the charge transfer (*Q*_t_) from or towards the GN nanosheet after the adsorption process according to the following equation:

(3) $$Q_{t}=Q_{combined\;GN}-Q_{isolated\;GN}$$

where $Q_{combinedGN}$ and $Q_{isolatedGN}$ are the charge of the GN nanosheet after and before the adsorption process, respectively. In addition, charge density difference (∆*ρ*) maps were plotted based on the following formula:

(4) $$∆\rho =\rho_{{cisPtX}_{2}\cdots GN}-\rho_{GN}-\rho_{{cisPtX}_{2}}$$

Visualization for Electronic and Structural Analysis (VESTA) package was utilized to generate ∆ρ maps [51]. The band structure, total density of states (TDOS), and projected density of states (PDOS) analyses were also conducted. To investigate the effect of the water solvent on the adsorption process, the environ code, which is available for Quantum ESPRESSO, was utilized with the self-consistent charge solvation model by using a dielectric constant of 78.3 [52]. The solvent effect ($E_{ads}^{solvent\;effect}$) on the adsorption process of the investigated complexes was evaluated as follows:

(5) $$E_{ads}^{solvent\;effect}=E_{ads}^{water}-E_{ads}^{vacuum}$$

where $E_{ads}^{water}$ and $E_{ads}^{vacuum}$ are the adsorption energies of the complex in water and vacuum media, respectively. Furthermore, the recovery time (τ) was also computed for the desorption process of the drug from the GN nanosheet based on the following equation:

(6) $$\tau =v_{}^{-1}\exp\left(-E_{ads}∕KT\right)$$

where $v_{}^{-1}$ stands for the attempt frequency with a value of 10^12^ s^−1^. *K* stands for the Boltzmann constant. T refers to the temperature, where the values of 295.15, 310.15, and 315.15 K were used for room, human body, and cancer cell temperatures, respectively. The computational approach adopted in this study was developed and successfully implemented in several previous reports [53,54,55,56].

## 3. Results and Discussion

### 3.1. Geometric Structures

A GN nanosheet was constructed, and all carbon atoms in the supercell were fully relaxed to obtain the equilibrium structure. Based on the relaxed structures, the lattice constant of the GN unit cell was *a* = 2.47 Å, which was in good agreement with the theoretical and experimental values for bulk graphite [57,58,59,60,61]. The GN nanosheet featured a symmetric carbon–carbon bond with a length of 1.42 Å, which produced three adsorption sites, namely, the top (T), hollow (H), and bridge (Br) sites.

### 3.2. Adsorption Energy Calculations

**Figure 3:**
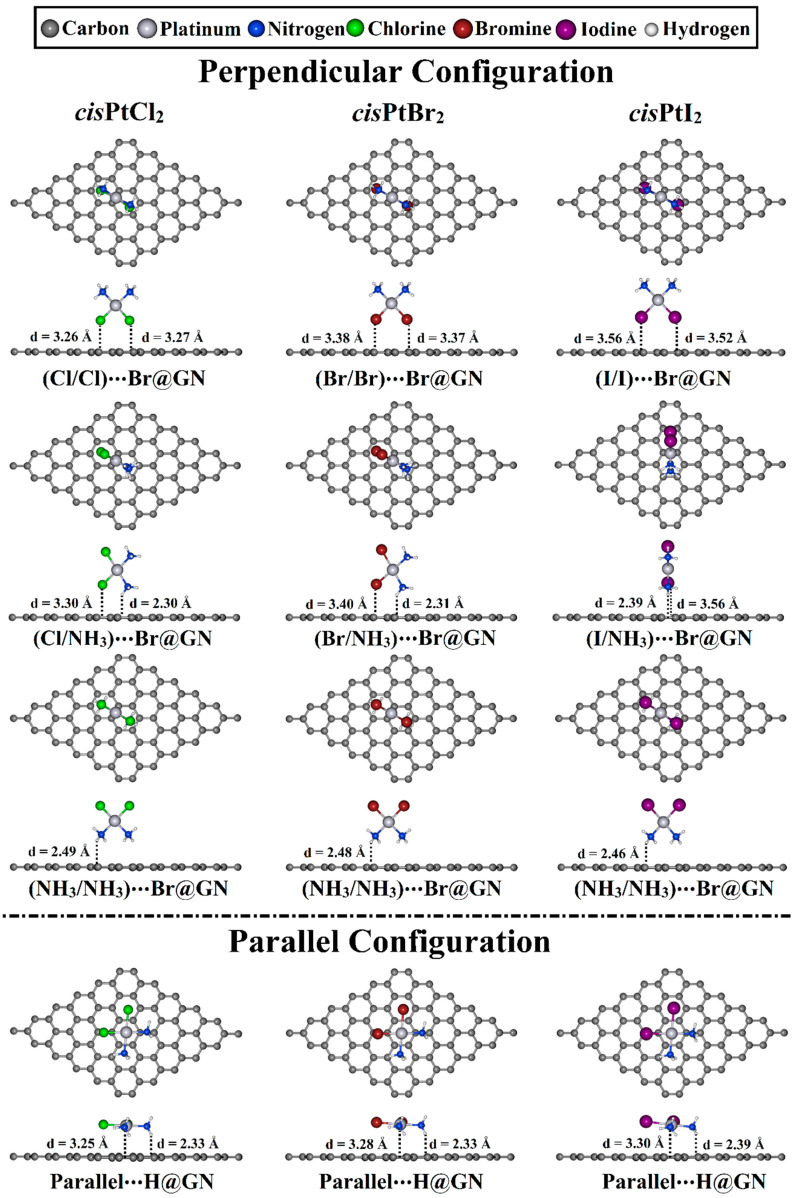
Top and side representations of the relaxed structures of *cis*PtX_2_⋯GN complexes (where X = Cl, Br, and I) in perpendicular and parallel configurations at the most favorable adsorption sites. The equilibrium distances are provided in Å.

The adsorption process of the *cis*PtX_2_ was explored at different adsorption sites on the GN nanosheet in perpendicular and parallel configurations (Figure 2). All of the constructed *cis*PtX_2_⋯GN complexes (where X = Cl, Br, and I) were fully relaxed, and their optimized structures are depicted in Figure S1. After that, the adsorption energies of the relaxed systems were computed, and their results are listed in Table 1. Based on the obtained adsorption energies, the structures of the most favorable *cis*PtX_2_⋯GN complexes are displayed in Figure 3.

As shown in Table 1, all relaxed *cis*PtX_2_⋯GN complexes showed negative adsorption energy values, demonstrating that *cis*PtX_2_ could be loaded onto the GN nanosheet. For the *cis*PtX_2_⋯GN complexes, it can be seen that the *cis*PtI_2_⋯GN complexes in the X/X orientation showed the most significant *E*_ads_ values, followed by the *cis*PtBr_2_⋯ and then *cis*PtCl_2_⋯GN complexes. For instance, the *E*_ads_ values of the *cis*PtI_2_⋯, *cis*PtBr_2_⋯, and *cis*PtCl_2_⋯T@GN were −10.68, −9.19, and −8.13 kcal/mol, respectively. Almost all *E*_ads_ values of the *cis*PtX_2_⋯GN complexes increased in the following order: *cis*PtX_2_⋯H@GN < ⋯T@GN < ⋯Br@GN. For example, the *E*_ads_ value of the *cis*PtI_2_⋯GN complexes were −10.68, −10.69, and −10.83 kcal/mol at H@GN, T@GN, and Br@GN sites, respectively (Table 1).

For the *cis*PtX_2_⋯GN complexes in the X/NH_3_ orientation, the negative *E*_ads_ values increased as the electronegativity of the halogen atom decreased. In this regard, the *cis*PtCl_2_⋯GN complexes showed the smallest negative *E*_ads_ values compared with the *cis*PtBr_2_⋯ and *cis*PtI_2_⋯GN complexes, with *E*_ads_ values of −9.89, −10.60, and −11.14 kcal/mol, respectively (Table 1).

In the NH_3_/NH_3_ orientation of the *cis*PtX_2_⋯GN complexes, the Br@GN site showed the most considerable adsorption energies compared with the T@GN and H@GN sites. For instance, the *E*_ads_ values of the *cis*PtCl_2_⋯Br@GN, ⋯T@GN, and ⋯H@GN complexes were −12.73, −12.60, and −12.27 kcal/mol, respectively (Table 1). The adsorption of the *cis*PtI_2_ on the GN nanosheet at the Br@GN site showed the largest *E*_ads_ value of −13.36 kcal/mol.

In the parallel configuration, the H@GN showed the most prominent adsorption energy with values of −22.76, −24.07, and −25.67 kcal/mol for *cis*PtCl_2_⋯, *cis*PtBr_2_⋯, and *cis*PtI_2_⋯H@GN, respectively.

To sum up, the negative *E*_ads_ values increased with the increase in the atomic weight of the halogen atoms in the following order: *cis*PtCl_2_⋯ < *cis*PtBr_2_⋯ < *cis*PtI_2_⋯GN complexes. The latter finding agreed with a finding of a previous study that reported the interaction strength decreased with the decrease in the atomic weight of the halogen atom [55]. The parallel configuration of the studied *cis*PtX_2_ molecules on the GN nanosheet had more significant adsorption energy than that in the perpendicular configuration. The Br@GN and H@GN sites were preferential for adsorbing the *cis*PtX_2_ in the perpendicular and parallel configurations, respectively. These results are in agreement with previously reported results for the most favorable conformation for the interaction of *cis*PtCl_2_ with different graphene models, in which the parallel configuration showed a more favorable binding with an average adsorption energy of 20 kcal/mol over different graphene models [37,38].

### 3.3. Frontier Molecular Orbital (FMO) Calculations

The energies of the highest occupied molecular orbitals (*E*_HOMO_), the lowest unoccupied molecular orbitals (*E*_LUMO_), and the energy gap (*E*_gap_) were evaluated to thoroughly reveal the impact of the adsorption process on the electronic characteristics of the investigated systems. The *E*_HOMO_, *E*_LUMO_, and *E*_gap_ values before and after the adsorption process are presented in Table 2 and Table 3, respectively. To understand the electron transfer regioselectivity of the studied molecules, the distributions of both HOMO and LUMO were generated for the isolated systems and the most favorable relaxed *cis*PtX_2_⋯GN complexes were determined (Figures S2 and S3).

From the summarized data in Table 2 and Table 3, the *E*_HOMO_, *E*_LUMO_, and *E*_gap_ values of the studied systems were observed with notable differences before and after the adsorption process. For example, in the parallel configuration, the *E*_HOMO_ values of the *cis*PtCl_2_⋯, *cis*PtBr_2_⋯, and *cis*PtI_2_⋯H@GN complexes were −2.155, −2.143, and −2.127 eV, respectively, whereas the pure GN nanosheet had an *E*_HOMO_ value of −2.355 eV (Table 2 and Table 3). Further, the *E*_gap_ values of the *cis*PtX_2_ molecules and GN nanosheet were changed after the adsorption process, demonstrating the occurrence of the adsorption. For instance, in the parallel configuration, the pure GN nanosheet had an *E*_gap_ value of 0.016 eV that was changed after the adsorption process to 0.026 eV in the case of the *cis*PtI_2_⋯H@GN complex (Table 2 and Table 3). Notably, the *E*_gap_ was denoted with small values, which demonstrated the feasibility of transferring the charge within the complex.

Looking at Figure S2, it can be seen that the HOMO orbitals of the *cis*PtX_2_ molecules were located on the halogen and platinum atoms, indicating that these atoms acted as electron donor sites in the adsorption process with the GN nanosheet. Furthermore, the LUMO orbitals were observed on the NH_3_ group of the *cis*PtX_2_ molecules, indicating the electron-accepting character of this group in the adsorption process. For the relaxed *cis*PtX_2_⋯GN complexes, the HOMO and LUMO orbitals were located on the carbon atoms of the GN nanosheet, while the LUMO orbitals were observed on the Pt atom, demonstrating its electron-accepting property (Figure S3).

### 3.4. Charge Transfer Calculations

Bader charge analysis is an effective tool for gaining better insight into charge transfer between the adsorbate and the substrate through adsorption processes [49,62]. Within the context of Bader charge analysis, the charge transfer differences (*Q*_t_) were determined for the relaxed *cis*PtX_2_⋯GN complexes in the perpendicular and parallel configurations (Table 1). Notably, the *Q*_t_ values had negative signs, indicating the charge transfer from the *cis*PtX_2_ to the GN nanosheet. In contrast to the negative *Q*_t_ values, the positive signs indicated that the charge shifted from the GN nanosheet to the adsorbed *cis*PtX_2_.

In the perpendicular configuration, all *cis*PtX_2_ in the X/X orientation had decreased electron-accepting properties, resulting in the following order: *cis*PtI_2_⋯ > *cis*PtBr_2_⋯ > *cis*PtCl_2_⋯GN complexes. For example, the *Q*_t_ values of the *cis*PtI_2_⋯, *cis*PtBr_2_⋯, and *cis*PtCl_2_⋯Br@GN complexes in the X/X orientation were 0.0375, 0.0252, and 0.0179 *e*, respectively (Table 1).

For the *cis*PtX_2_⋯GN complexes in the X/NH_3_ orientation, negative *Q*_t_ values were observed for the *cis*PtCl_2_⋯GN complexes, indicating the ability of the GN nanosheet to accept the charge from the *cis*PtCl_2_ drug. Compared to the *cis*PtCl_2_⋯GN complexes, the *cis*PtBr_2_⋯ and *cis*PtI_2_⋯GN complexes had positive *Q*_t_ values, demonstrating the electron-accepting character of the *cis*PtBr_2_ and *cis*PtI_2_. As an example, the *Q*_t_ values of the *cis*PtCl_2_⋯, *cis*PtBr_2_⋯, and *cis*PtI_2_⋯Br@GN complexes in the X/NH_3_ orientation were −0.0056, 0.0008, and 0.0124 *e*, respectively (Table 1).

In the NH_3_/NH_3_ orientation, the adsorption of the *cis*PtCl_2_ and *cis*PtBr_2_ on the GN nanosheet led to a transfer of the charge from the adsorbate to the substrate, which was indicated by the negative *Q*_t_ values. In comparison, the *cis*PtI_2_ acted as an electron donor within the *cis*PtI_2_⋯GN complexes, giving positive *Q*_t_ values (Table 1). Notable electron-donating properties were observed for *cis*PtCl_2_ and *cis*PtBr_2_ and disappeared for *cis*PtI_2_ within the adsorption process in the NH_3_/NH_3_ orientation. For instance, the *Q*_t_ values of the *cis*PtCl_2_⋯, *cis*PtBr_2_⋯, and *cis*PtI_2_⋯Br@GN were −0.0159, −0.0028, and 0.0250 *e*, respectively (Table 1).

In the parallel configuration, almost all of the *Q*_t_ values of the *cis*PtX_2_⋯GN complexes had positive signs, revealing the electron-donating character of the GN nanosheet. In this regard, the ability of *cis*PtX_2_ to gain the charge from the GN nanosheet increased with the increase in the atomic weight of the halogen atom. For example, the *cis*PtCl_2_⋯, *cis*PtBr_2_⋯, and *cis*PtI_2_⋯H@GN complexes had positive *Q*_t_ with values of 0.0042, 0.0156, and 0.0312 *e*, respectively (Table 1).

**Figure 4:**
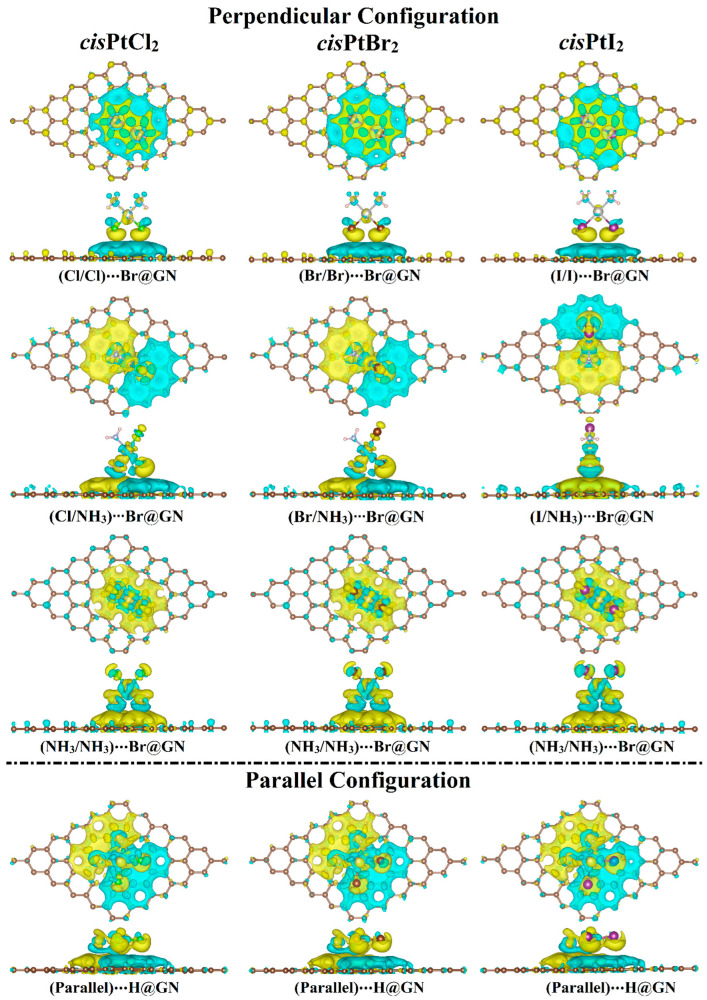
Charge density difference (∆*ρ*) maps of the most favorable *cis*PtX_2_⋯GN complexes (where X = Cl, Br, and I) in the perpendicular and parallel configurations. Electron accumulation and depletion sites are indicated by yellow- and cyan-colored regions, respectively. Pale brown, silver, pink, gray, green, dark brown, and violet balls refer to carbon, platinum, hydrogen, nitrogen, chloride, bromide, and iodide atoms, respectively.

Following the Bader charge analysis, the charge density difference (Δ*ρ*) maps were generated for the most favorable *cis*PtX_2_⋯GN complexes to evaluate the distribution of charge (Figure 4). According to the Δ*ρ* maps, the amount of the accumulated (i.e., positive) and depleted (i.e., negative) charge agreed with the *Q*_t_ results (Table 1). For example, in the X/X orientation, adsorption of *cis*PtX_2_ on GN nanosheet showed electron-accepting properties in the perpendicular and parallel configurations, as confirmed by the accumulated charge region (yellow color) below the *cis*PtX_2_ (Figure 4). In line with the *E*_ads_ findings, the parallel configuration of the *cis*PtX_2_ ⋯GN complexes showed that the largest amount of charge was accumulated in a region distributed over the complexes. However, it can be seen that in the X/NH_3_ orientation, the charge-depleted region was observed below the NH_3_ part, and the charge-accumulated region was noted below the X part, as shown by the cyan and yellow colors, respectively. The latter observation indicated that the X atoms had the dominant contribution to the adsorption process of *cis*PtX_2_ on the GN nanosheet.

Based on the Bader charge outcomes, the *cis*PtI_2_ behaved as an electron acceptor through the adsorption process on the GN nanosheet in both the perpendicular and parallel configurations. The adsorption of the *cis*PtBr_2_ on the GN nanosheet resulted in gaining the charge from the GN nanosheet in both configurations, except for in the NH_3_/NH_3_ orientation in the perpendicular configuration. Furthermore, the electron-accepting character of the GN nanosheet decreased as the electronegativity of the X atom decreased.

### 3.5. Band Structure Calculations

**Figure 5:**
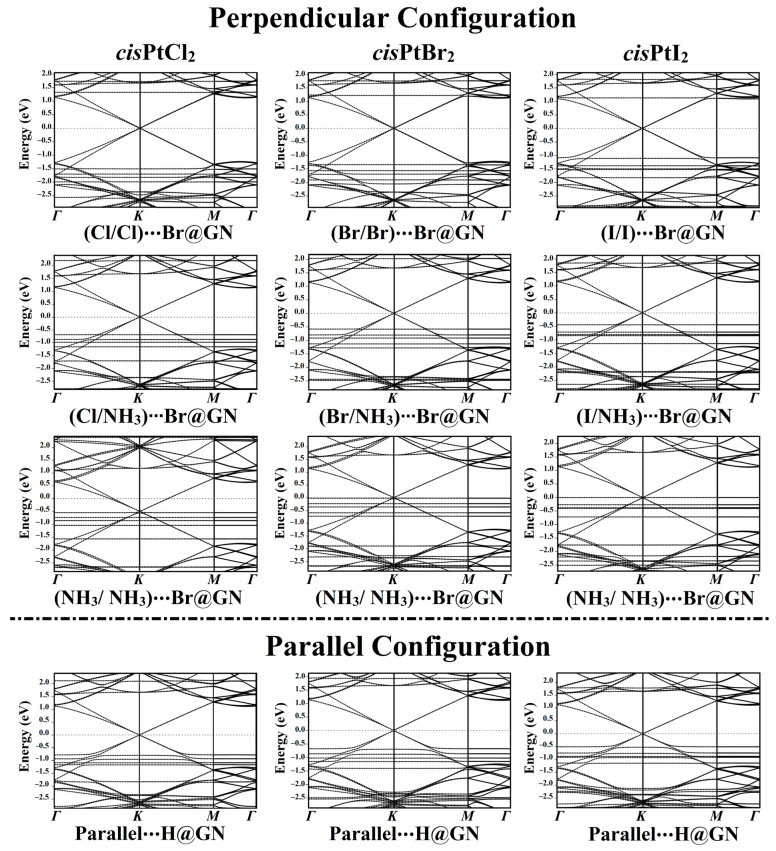
Electronic band structures of the most favorable *cis*PtX_2_⋯GN complexes (where X = Cl, Br, and I) in the perpendicular and parallel configurations along the high-symmetry path of *Γ*-*K*-*M*-*Γ*. Energy values are displayed relative to the Fermi energy, and the Fermi level sets at zero.

In order to ascertain how the adsorbed *cis*PtX_2_ affected the electronic properties of the GN nanosheet, electronic band structure calculations were performed for the GN nanosheet before and after the adsorption process (Figure 5 and Figure S4a).

According to the *E*_ads_ findings, the electronic band structures were plotted for the most favorable *cis*PtX_2_⋯GN complexes in the perpendicular and parallel configurations (Figure 5). As shown in Figure 5, all of the band structure plots demonstrated that the adsorption of *cis*PtX_2_ on the GN nanosheet affected the electronic characteristics of the pure GN surface.

In the perpendicular configuration of the studied complexes in the X/X orientation, new bands appeared for the *cis*PtX_2_⋯Br@GN complexes, highlighting the contribution of the *cis*PtX_2′_s bands with those of the pure GN nanosheet. In the *cis*PtCl_2_⋯Br@GN complex, additional bands appeared at −1.53, −1.70, −1.80, and −2.00 eV in the valence region, while in the conduction region, new bands appeared at 1.75 and 1.35 eV (Figure 5). The adsorption of the *cis*PtBr_2_ at the Br@GN site resulted in the appearance of new valence bands at −1.20, −1.55, −1.62, and −2.08 eV, while additional bands in the conduction region were observed at 1.75 and 1.23 eV. Obviously, the valence and conduction bands in the *cis*PtI_2_⋯Br@GN complex were shifted toward the Fermi level, announcing the significant adsorption process of the *cis*PtI_2_ on the GN nanosheet. The latter observation confirmed the significant adsorption of the *cis*PtI_2_ on the GN nanosheet, which was compatible with the *E*_ads_ findings (Table 1).

New bands were observed in the band structures of the *cis*PtX_2_⋯GN complexes in the X/NH_3_ orientation. For instance, in the *cis*PtBr_2_⋯Br@GN complex, many bands in the valence region were noted between −0.58 and −2.50 eV. In line with the adsorption energy affirmations, the band structures showed that the *cis*PtI_2_⋯GN complexes were preferable, as revealed by the bands that shifted toward the Fermi level (Figure 5).

From the band structure plots of the *cis*PtX_2_⋯Br@GN complexes in the NH_3_/NH_3_ orientation, it can be seen that additional valence bands appeared. For example, for the *cis*PtI_2_⋯Br@GN complex, new bands were observed at −0.28, −0.39, −0.40, −0.72, −1.75, −2.35, and −2.50 eV. Notably, all bands moved toward the Fermi level, particularly the valence bands of the *cis*PtI_2_⋯Br@GN complex in the NH_3_/NH_3_ orientation, which reached 0.00 eV at the Fermi level (Figure 5).

In addition, the adsorption of the *cis*PtX_2_ on the GN nanosheet in the parallel configuration led to the appearance of new bands in the valence and conduction regions. For example, the band structure of the *cis*PtI_2_⋯H@GN complex showed new valence bands at −2.25 and −2.30 eV, while new conduction bands appeared at 0.50, 0.75, 0.88, and 1.15 eV.

Summing up, the adsorption of the *cis*PtX_2_ on the GN nanosheet in the perpendicular and parallel configurations affected the band structure of the GN nanosheet. In line with the *E*_ads_ and *Q*_t_ findings, the band structure plots revealed the most favorable adsorption process of the *cis*PtI_2_ on the GN nanosheet. In addition, the presence of the Dirac point on the GN nanosheet after the adsorption process indicated the physical adsorption of the *cis*PtX_2_ on the GN nanosheet.

### 3.6. Density of States (DOS) Calculations

**Figure 6:**
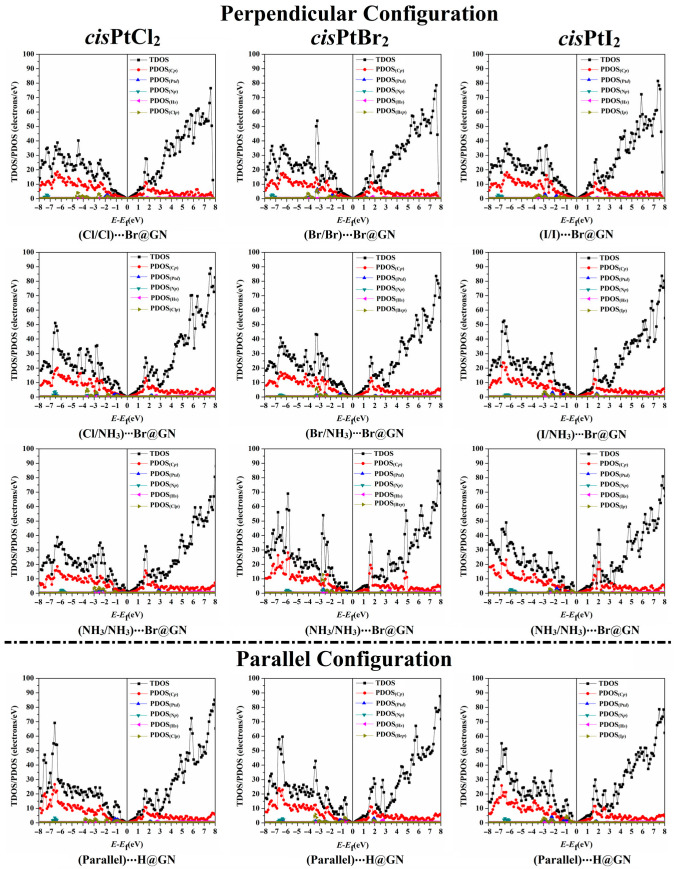
The TDOS and PDOS plots of the most favorable *cis*PtX_2_⋯GN complexes (where X = Cl, Br, and I) in the perpendicular and parallel configurations. C*_p_*, N*_p_*, Cl*_p_*, Br*_p_*, and I*_p_* represent the contributions of the *p*-orbital of carbon, nitrogen, chloride, bromide, and iodide atoms, respectively. Pt*_d_* and H*_s_* represent the *d*-orbital and *s*-orbital of platinum and hydrogen atoms, respectively.

To describe the influence of the adsorption of the *cis*PtX_2_ on the electronic characteristics of the GN nanosheet, the TDOS and PDOS were generated for pure and combined GN nanosheets (Figure S4b). Figure 6 illustrates the TDOS and PDOS analyses for the most favorable *cis*PtX_2_⋯GN complexes in the perpendicular and parallel configurations.

As depicted in Figure 6, it was observed that the adsorption process mainly originated from the contributions of the X*_p_*, C*_p_*, N*_p_*, and Pt*_d_* of the *cis*PtX_2_ with the C*_p_* of the GN nanosheet. In contrast, the H*_s_* of the *cis*PtX_2_ showed a small effect on the adsorption process. For instance, the PDOS plot of the *cis*PtCl_2_⋯Br@GN complex in both the perpendicular and parallel configurations demonstrated the contribution of the Cl*_p_* to the adsorption process, which appeared in the valence region from −4.50 to −1.00 eV. In addition, the participation of N*_p_* and Pt*_d_* in the adsorption process of the *cis*PtCl_2_ on the GN nanosheet was detected in the valence region at energies ranging from −7.40 to −5.90 eV and from −7.20 to −0.60 eV, respectively (Figure 6). In both the perpendicular and parallel configurations, the appearance of new peaks demonstrated the occurrence of the adsorption process of the *cis*PtX_2_ on the GN nanosheet, which affirmed the findings of the band structure. At the Fermi level, the Dirac point with zero DOS confirmed that the adsorption of *cis*PtX_2_ on the GN nanosheet had a small effect on the electronic properties of the pure GN nanosheet.

### 3.7. Recovery Time

Recovery time (τ) calculations are necessary to comprehend the desorption process of the *cis*PtX_2_ from the GN nanosheet. Therefore, τ was evaluated at three different temperatures. The findings on τ for the most favorable *cis*PtX_2_⋯GN complexes (where X = Cl, Br, and I) in the perpendicular and parallel configurations are listed in Table 4.

According to the data in Table 4, τ had a direct correlation with the *E*_ads_ findings, showing that as the negative *E*_ads_ value increased, τ increased, and the desorption process became more difficult. For example, the *cis*PtI_2_⋯H@GN complex in the parallel configuration had the most prominent negative *E*_ads_ with a value of −25.67 kcal/mol and the longest τ of 61.63 × 10^8^, 11.56 × 10^8^, and 5.97 × 10^8^ ms at the room, human body, and cancer cell temperatures, respectively. The τ values showed a clear decrease with increasing temperature; for instance, the τ values of the *cis*PtCl_2_⋯Br@GN complexes were 12.30 × 10^−4^, 7.15 × 10^−4^, and 5.77 × 10^−4^ ms at the room, human body, and cancer cell temperatures, respectively (Table 4). Therefore, the desorption process at the temperature of cancer cells showed the fastest τ.

### 3.8. Solvent Effects

In order to hypothesize about the influence of solvent on the adsorption process of the *cis*PtX_2_ on the GN nanosheet, the adsorption energy was assessed in the presence of a water medium. The solvent effect ($E_{ads}^{solvent\;effect}$) energy was calculated for the most favorable *cis*PtX_2_⋯GN configurations by subtracting the adsorption energies in the vacuum medium from those in the water solvent. The computed $E_{ads}^{water}$ and $E_{ads}^{solvent\;effect}$ values are tabulated in Table 5.

According to Table 5, negative $E_{ads}^{water}$ values demonstrated that the GN nanosheet had the potential to adsorb the *cis*PtX_2_ in a water solvent within the perpendicular and parallel configurations. It can be seen that the parallel configuration of the *cis*PtX_2_⋯H@GN complexes showed the most significant negative $E_{ads}^{water}$ with values of −18.21, −20.02, and −22.40 kcal/mol for *cis*PtCl_2_⋯, *cis*PtBr_2_⋯, and *cis*PtI_2_⋯H@GN, respectively.

Generally, the obtained results showed that the most favorable *cis*PtX_2_⋯GN complexes in the water solvent had lower negative *E*_ads_ values compared to those in the vacuum medium. For instance, the *cis*PtCl_2_⋯H@GN complex had negative *E*_ads_ values of −22.76 and −18.21 kcal/mol in the vacuum and water media, respectively. The latter observation revealed the occurrence of physical adsorption between the *cis*PtX_2_ and the GN nanosheet in the water medium.

## 4. Conclusions

To gain a better insight into the use of GN nanosheets as nanocarriers for anticancer drugs, the adsorption behavior of *cis*PtCl_2_ and its analogs (*cis*PtX_2_, where X = Br, and I) on a GN nanosheet in the perpendicular and parallel configurations was investigated. Based on the findings, the largest negative *E*_ads_ values were observed for the parallel configuration of the *cis*PtX_2_⋯GN complexes with values of up to −25.67 kcal/mol. In the perpendicular configuration of the *cis*PtX_2_⋯GN complexes, three possible orientations were observed, namely, X/X, X/NH_3_, and NH_3_/NH_3_. The NH_3_/NH_3_ orientation had the greatest negative *E*_ads_ values compared to the other orientations. For instance, the *E*_ads_ values of the *cis*PtCl_2_⋯T@GN complexes in the X/X, X/NH_3_, and NH_3_/NH_3_ orientations were −8.13, −9.70, and −12.60 kcal/mol, respectively. Remarkably, the negative *E*_ads_ values decreased by increasing the electronegativity of the halogen atoms within the *cis*PtX_2_⋯GN complexes in the following order: *cis*PtI_2_⋯ > *cis*PtBr_2_⋯ > *cis*PtCl_2_⋯GN. The Br@GN site showed the largest negative *E*_ads_ values in the perpendicular configuration for the *cis*PtX_2_⋯GN complexes, while the H@GN was the most favorable site in the parallel configuration. Based on FMO findings, changes in the *E*_HOMO_, *E*_LUMO_, and *E*_gap_ values of the GN nanosheet were noticed after the adsorption process. According to the Bader charge outlines, the *cis*PtI_2_ exhibited an electron-accepting character through the adsorption process on the GN nanosheet in both configurations. The appearance of new bands and peaks in the band structure and the DOS plots affirmed the occurrence of the adsorption process of the *cis*PtX_2_ on the GN nanosheet. The solvent effect results demonstrated that the *cis*PtX_2_ could be adsorbed on the GN nanosheet in a water solvent via a physical adsorption process. The *cis*PtI_2_⋯GN complexes with the largest negative *E*_ads_ values had the longest recovery time for the desorption process in the parallel configuration of up to 61.63 × 10^8^ ms at 298.15 K. The outcomes of this work will contribute to a better understanding of the utilization of GN nanosheets in drug delivery applications for anticancer drugs. Developing a better understanding of the adsorption process of cisplatin, its analogs, and other reported drug molecules on GN surfaces opens the door for more efficiently designed GN-based nanocarriers and provides the possibility of co-adsorption of more than one active drug molecule.

## Figures and Tables

**Table 1:** Adsorption energy (*E*_ads_, kcal/mol) of *cis*PtX_2_ (where X = Cl, Br, and I) on the GN nanosheet at all adsorption sites. The charge transfer (*Q*_t_) of the GN nanosheet before and after the adsorption process is given in *e*.

Orientation ^a^	Adsorption Site ^b^	*cis*PtCl_2_		*cis*PtBr_2_		*cis*PtI_2_	
		*E*_ads_ (kcal/mol)	*Q_t_* ^c^ (*e*)	*E*_ads_ (kcal/mol)	*Q_t_* ^c^ (*e*)	*E*_ads_ (kcal/mol)	*Q_t_* ^c^ (*e*)
**Perpendicular Configuration**							
X/X⋯GN	T	−8.13	0.0165	−9.19	0.0244	−10.69	0.0369
	Br	−8.32	0.0179	−9.38	0.0252	−10.83	0.0375
	H	−7.93	0.0147	−9.10	0.0239	−10.68	0.0385
X/NH_3_⋯GN	T	−9.70	−0.0051	−10.15	0.0001	−10.99	0.0102
	Br	−9.89	−0.0056	−10.60	0.0008	−11.14	0.0124
	H	−9.33	−0.0054	−9.85	0.0003	−11.12	0.0090
NH_3_/NH_3_⋯GN	T	−12.60	−0.0189	−12.73	−0.0105	−13.09	0.0221
	Br	−12.73	−0.0159	−13.01	−0.0028	−13.36	0.0250
	H	−12.27	−0.0215	−12.39	−0.0091	−12.84	0.0234
**Parallel Configuration**							
Parallel⋯GN	T	−22.56	−0.0008	−23.98	0.0147	−25.24	0.0298
	Br	−22.67	0.0014	−24.03	0.0125	−25.61	0.0310
	H	−22.76	0.0042	−24.07	0.0156	−25.67	0.0312

^a^ Possible orientations for adsorption of *cis*PtX_2_ on the GN nanosheet are shown in Figure S1. ^b^ Adsorption sites on the surface of the GN nanosheet (see Figure 1). ^c^ *Q*_t_ was evaluated according to Equation (3).

**Table 2:** The energies of the highest occupied molecular orbitals (*E*_HOMO_, eV), the lowest unoccupied molecular orbitals (*E*_LUMO_, eV), and the energy gap (*E*_gap_, eV) of the cisPtX_2_ molecules and the GN nanosheet before the adsorption process.

System	*E*_HOMO_ (eV)	*E*_LUMO_ (eV)	*E*_gap_ (eV)
GN Nanosheet	−2.355	−2.339	0.016
*cis*PtCl_2_	−4.824	−1.992	2.831
*cis*PtBr_2_	−4.720	−2.161	2.559
*cis*PtI_2_	−4.547	−2.313	2.235

**Table 3:** The energies of the highest occupied molecular orbitals (*E*_HOMO_, eV), the lowest unoccupied molecular orbitals (*E*_LUMO_, eV), and the energy gap (*E*_gap_, eV) of the most favorable relaxed *cis*PtX_2_⋯GN complexes.

Orientation ^a^	System ^b^	*E*_HOMO_ (eV)	*E*_LUMO_ (eV)	*E*_gap_ (eV)
**Perpendicular Configuration**				
X/X⋯Br@GN	*cis*PtCl_2_	−1.566	−1.557	0.009
	*cis*PtBr_2_	−1.571	−1.562	0.009
	*cis*PtI_2_	−1.571	−1.561	0.009
X/NH_3_⋯Br@GN	*cis*PtCl_2_	−2.173	−2.158	0.015
	*cis*PtBr_2_	−2.173	−2.159	0.015
	*cis*PtI_2_	−2.141	−2.128	0.014
NH_3_/NH_3_⋯Br@GN	*cis*PtCl_2_	−2.672	−2.657	0.015
	*cis*PtBr_2_	−2.626	−2.612	0.015
	*cis*PtI_2_	−2.528	−2.523	0.005
**Parallel Configuration**				
Parallel⋯H@GN	*cis*PtCl_2_	−2.155	−2.129	0.025
	*cis*PtBr_2_	−2.143	−2.118	0.025
	*cis*PtI_2_	−2.127	−2.101	0.026

^a^ Possible orientations for adsorption of *cis*PtX_2_ on the GN nanosheet are depicted in Figure S1. ^b^ The most favorable relaxed *cis*PtX_2_⋯GN complexes are depicted in Figure 3.

**Table 4:** Recovery time (τ) for the most favorable *cis*PtX_2_⋯GN complexes (where X = Cl, Br, and I) in the perpendicular and parallel configurations at room (298.15 K), human body (310.15 K), and cancer cell (315.15 K) temperatures.

Orientation ^a^	*cis*PtX_2_⋯GN Complexes	Recovery Time (ms)		
		T (298.15 K)	T (310.15 K)	T (315.15 K)
**Perpendicular Configuration**				
X/X	*cis*PtCl_2_⋯Br@GN	12.30 × 10^−4^	7.15 × 10^−4^	5.77 × 10^−4^
	*cis*PtBr_2_⋯Br@GN	7.34 × 10^−3^	3.90 × 10^−3^	3.13 × 10^−3^
	*cis*PtI_2_⋯Br@GN	8.45 × 10^−2^	4.17 × 10^−2^	3.16 × 10^−2^
X/NH_3_	*cis*PtCl_2_⋯Br@GN	17.34 × 10^−3^	9.10 × 10^−3^	7.05 × 10^−3^
	*cis*PtBr_2_⋯Br@GN	5.74 × 10^−2^	2.80 × 10^−2^	2.19 × 10^−2^
	*cis*PtI_2_⋯Br@GN	14.26 × 10^−2^	6.90 × 10^−2^	5.18 × 10^−2^
NH_3_/NH_3_	*cis*PtCl_2_⋯Br@GN	2.08	0.90	0.65
	*cis*PtBr_2_⋯Br@GN	3.33	1.42	1.02
	*cis*PtI_2_⋯Br@GN	6.01	2.52	1.79
**Parallel Configuration**				
Parallel	*cis*PtCl_2_⋯H@GN	45.68 × 10^6^	10.36 × 10^6^	5.77 × 10^6^
	*cis*PtBr_2_⋯H@GN	41.56 × 10^7^	8.65 × 10^7^	4.66 × 10^7^
	*cis*PtI_2_⋯H@GN	61.63 × 10^8^	11.56 × 10^8^	5.97 × 10^8^

^a^ Possible orientations for the adsorption of *cis*PtX_2_ on the GN nanosheet are presented in Figure S1.

**Table 5:** Adsorption energy in the water medium ($E_{ads}^{water}$, kcal/mol) and the solvent effect energy ($E_{ads}^{solvent\;effect}$, kcal/mol) for the most favorable *cis*PtX_2_⋯GN complexes (where X = Cl, Br, and I) in the perpendicular and parallel configurations.

Orientation ^a^	*cis*PtX_2_⋯GN Complexes	$E_{ads}^{water}$ (kcal/mol)	$E_{ads}^{solvent\;effect}$ (kcal/mol)
**Perpendicular Configuration**			
X/X	*cis*PtCl_2_⋯Br@GN	−8.10	0.2240
	*cis*PtBr_2_⋯Br@GN	−10.14	−0.7594
	*cis*PtI_2_⋯Br@GN	−12.92	−2.0973
X/NH_3_	*cis*PtCl_2_⋯Br@GN	−9.24	0.6490
	*cis*PtBr_2_⋯Br@GN	−10.40	0.2039
	*cis*PtI_2_⋯Br@GN	−11.57	−0.4303
NH_3_/NH_3_	*cis*PtCl_2_⋯Br@GN	−8.94	3.7919
	*cis*PtBr_2_⋯Br@GN	−9.02	3.9848
	*cis*PtI_2_⋯Br@GN	−9.14	4.0657
**Parallel Configuration**			
Parallel	*cis*PtCl_2_⋯H@GN	−18.21	4.5553
	*cis*PtBr_2_⋯H@GN	−20.02	4.0526
	*cis*PtI_2_⋯H@GN	−22.40	3.2723

^a^ Possible orientations for the adsorption of *cis*PtX_2_ on the GN nanosheet are depicted in Figure S1.

## Data Availability

Data will be made available on request.

## Supplementary Materials

The following supporting information can be downloaded at: https://www.mdpi.com/article/10.3390/pharmaceutics15061640/s1, Figure S1: Top and side views of all the relaxed structures for *cis*PtX_2_⋯GN complexes (where X = Cl, Br, and I) in the perpendicular and parallel configurations at all adsorption sites. The equilibrium distances (d) are presented in Å.; Figure S2: Distributions of the HOMO and LUMO for the GN nanosheet and *cis*PtX_2_ molecules before the adsorption process.; Figure S3: Distributions of the HOMO and LUMO for the most favorable relaxed *cis*PtX_2_⋯GN complexes in the parallel configuration.; Figure S4: (a) Band structure of the pure GN nanosheet along high-symmetry points of the Brillouin zone; (b) total and projected density of states (TDOS/PDOS) for the pure GN nanosheet. The contribution of the *p*-orbital of carbon atoms is indicated by C*_p_*. The Fermi level is located at zero energy.
