# Supplementary material for: On the Use of Graphene Nanosheets for Drug Delivery: A Case Study of Cisplatin and Some of Its Analogs
Source: Pharmaceutics. 2023 Jun 1;15(6):1640. doi: 10.3390/pharmaceutics15061640 (PMC10301707; doi:10.3390/pharmaceutics15061640)
Supplement: Supplementary file 1 [file pharmaceutics-15-01640-s001.zip › pharmaceutics-2416151-supplementary.pdf]

Supplementary Information

On the Use of Graphene Nanosheets for the Drug Delivery: A Case Study of Cisplatin and some of its Analogs

Mahmoud A. A. Ibrahim[1,2,*], Manar H. A. Hamad[1], Amna H. M. Mahmoud[1], Gamal A. H. Mekhemer[1], Shaban R. M. Sayed[3], Mohamed K. Abd El-Rahman[4], Peter A. Sidhom[5], Eslam Dabbish[6], and Tamer Shoeib[6,*]

[1] Computational Chemistry Laboratory, Chemistry Department, Faculty of Science, Minia University, Minia 61519, Egypt; m.ibrahim@compchem.net; m.hamad@compchem.net; a.mahmoud@compchem.net; gmekhemer@mu.edu.eg.

[2] School of Health Sciences, University of KwaZulu-Natal, Westville, Durban 4000, South Africa

[3] Department of Botany and Microbiology, College of Science, King Saud University, P.O. Box 2455, Riyadh 11451, Saudi Arabia; shmohamed@ksu.edu.sa

[4] Department of Chemistry and Chemical Biology, Harvard University, 12 Oxford Street, Cambridge, MA 02138, USA; kabdelazim@gmwgroup.harvard.edu

[5] Department of Pharmaceutical Chemistry, Faculty of Pharmacy, Tanta University, Tanta 31527, Egypt; peter.ayoub@pharm.tanta.edu.eg.

[6] Department of Chemistry, The American University in Cairo, New Cairo 11835, Egypt; emoustafa@aucegypt.edu; t.shoeib@aucegypt.edu

* Correspondence: m.ibrahim@compchem.net (M.A.A.I.) and t.shoeib@aucegypt.edu (T.S.).

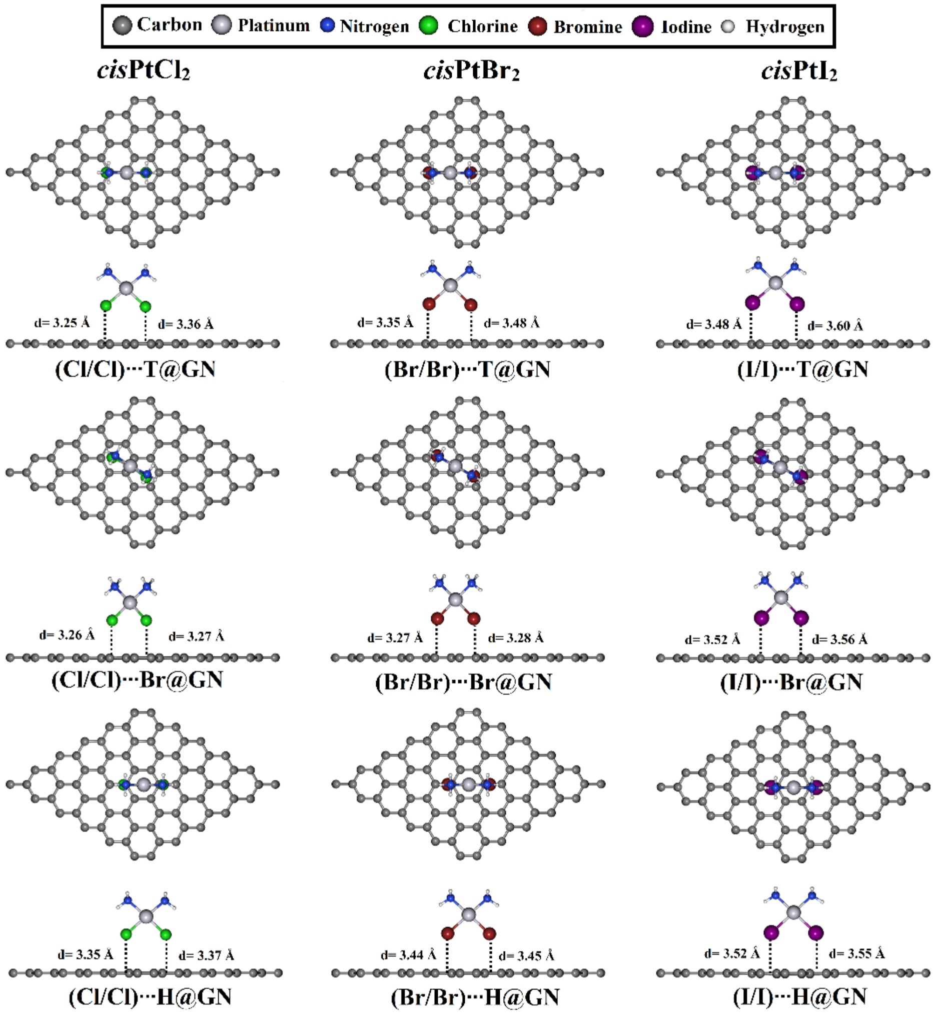

Figure S1. Top and side views of all the relaxed structures for *cis*PtX_2···GN complexes (where X = Cl, Br, and I) in perpendicular and parallel configurations at all adsorption sites. The equilibrium distances (d) are presented in Å.

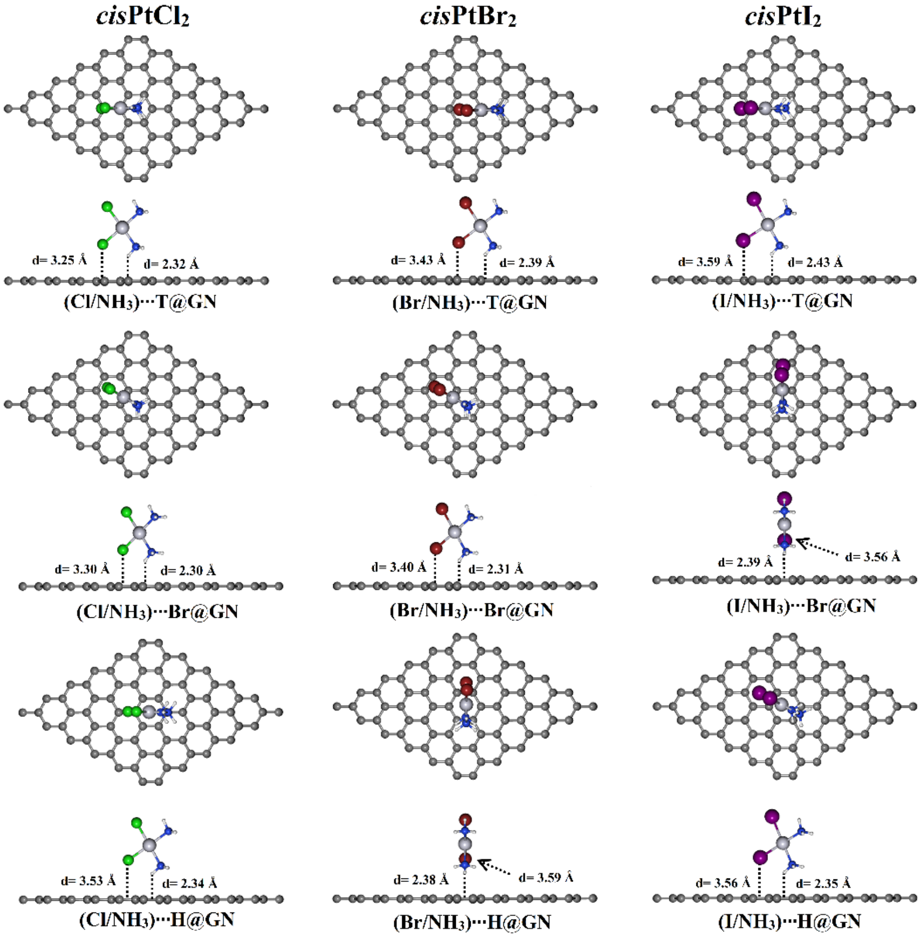

Figure S1. Continued**.**

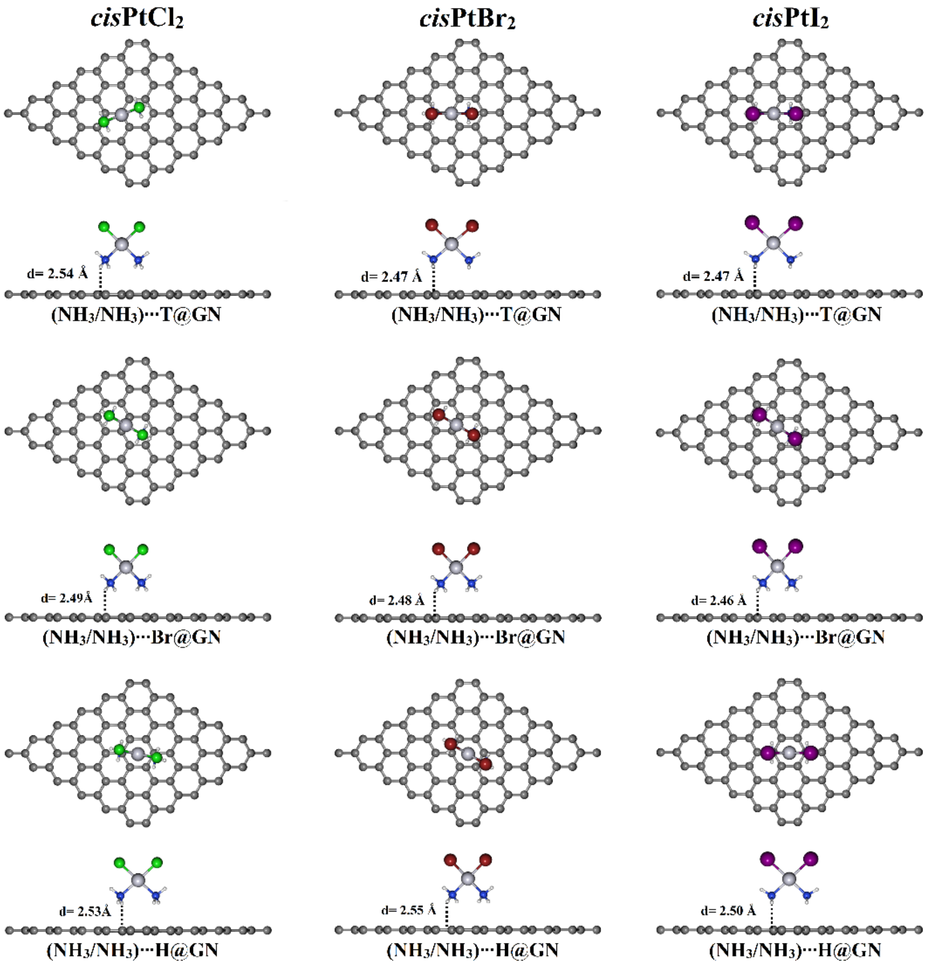

Figure S1. Continued**.**

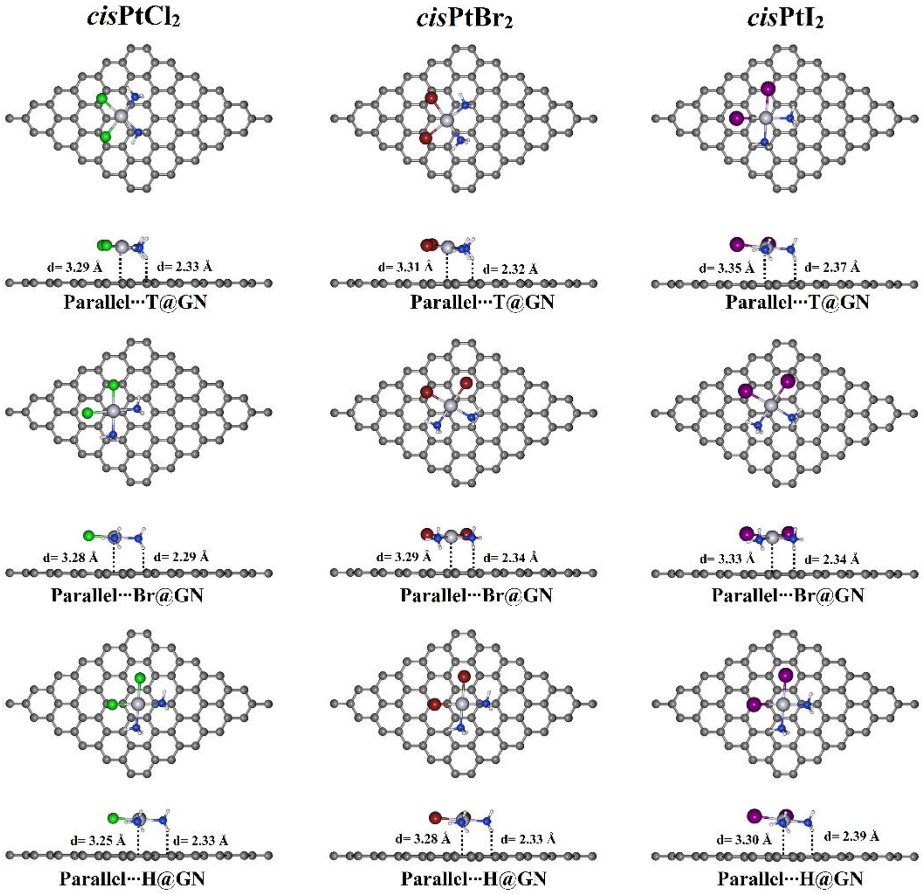

Figure S1. Continued**.**

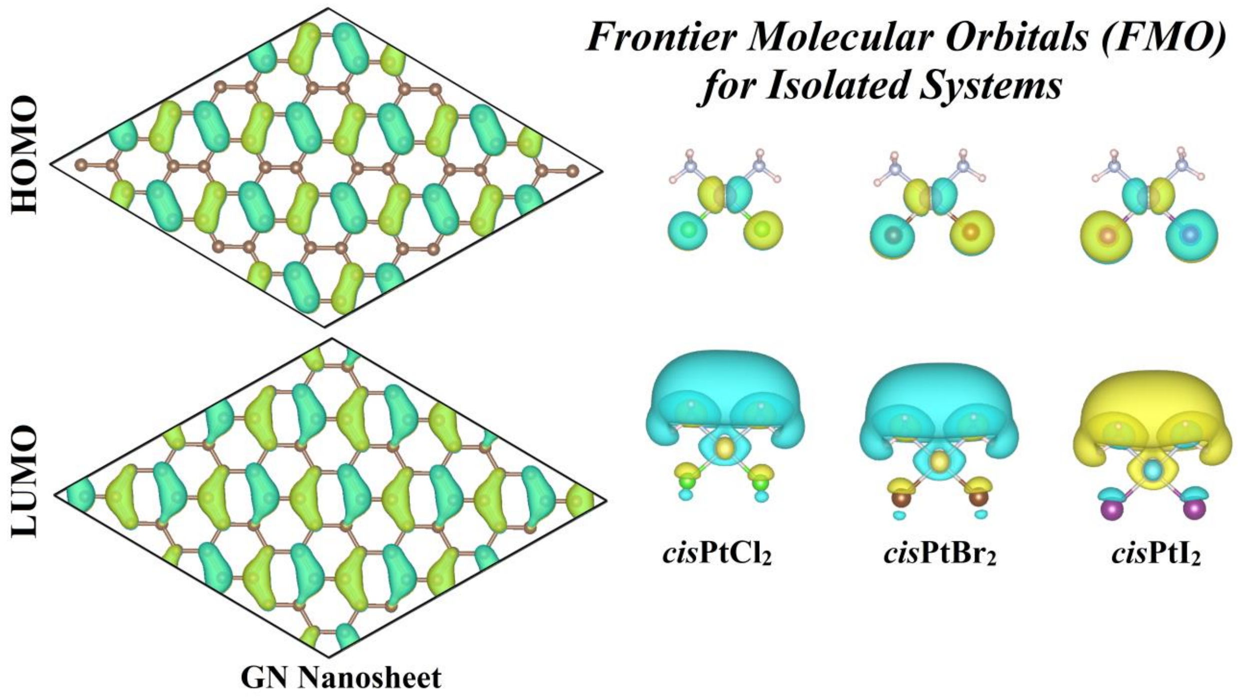

Figure S2. Distributions of HOMO and LUMO for the GN nanosheet and *cis*PtX_2 molecules before the adsorption process. Pale brown, silver, pink, gray, green, dark brown, and violet balls refer to carbon, platinum, hydrogen, nitrogen, chloride, bromide, and iodide atoms, respectively.

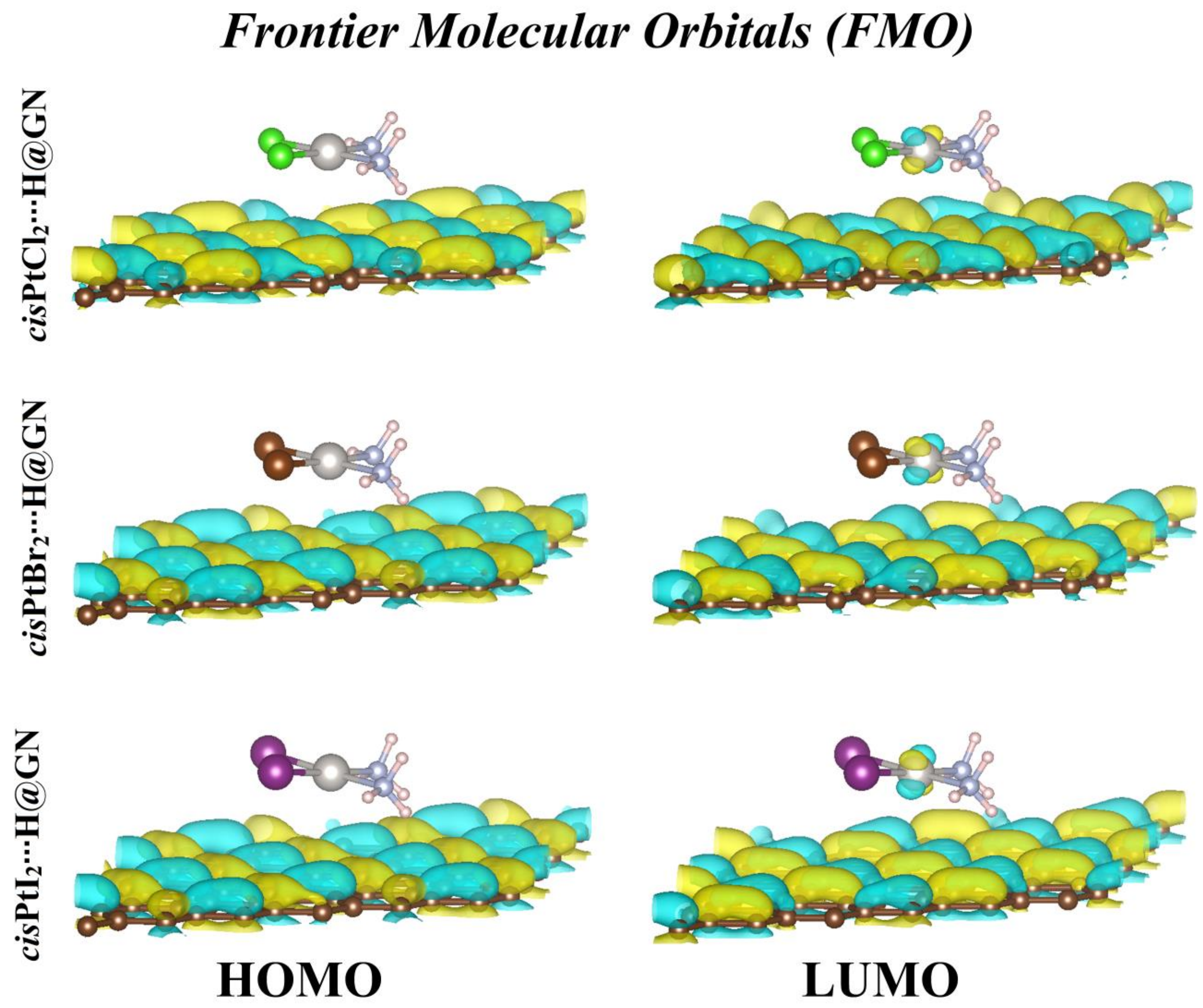

Figure S3. Distributions of HOMO and LUMO for the most favorable relaxed *cis*PtX_2···GN complexes within the parallel configuration. Pale brown, silver, pink, gray, green, dark brown, and violet balls refer to carbon, platinum, hydrogen, nitrogen, chloride, bromide, and iodide atoms, respectively.

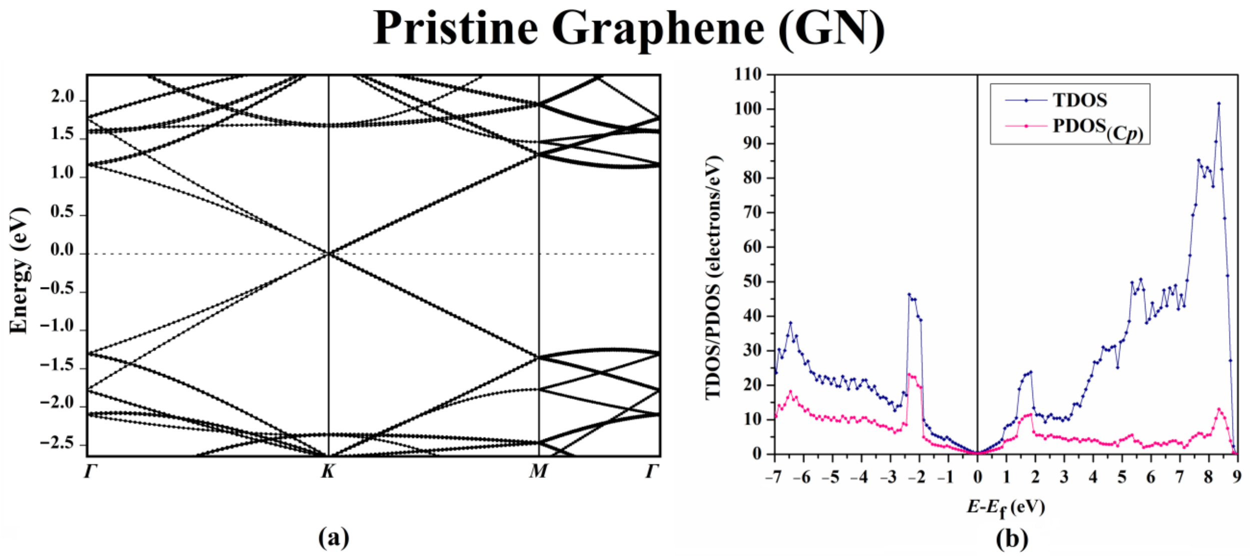

Figure S4. (a) Band structure of the pure GN nanosheet along high symmetry points of Brillouin zone, (b) Total and projected density of state (TDOS/PDOS) for the pure GN nanosheet, the contribution of *p*-orbital for carbon atom displayed by C_p. the Fermi level is located at zero energy.
